# Where are the children? Case finding in 5–14-year-olds living with HIV in Johannesburg

**DOI:** 10.4102/sajhivmed.v23i1.1378

**Published:** 2022-04-14

**Authors:** Jackie L. Dunlop, Carol L. Tait, Moyahabo Mabitsi, Kate Rees

**Affiliations:** 1 Anova Health Institute, Johannesburg, South Africa; 2 Division of Child Health, Faculty of Health Sciences, University of the Witwatersrand, Johannesburg, South Africa; 3 Department of Public Health, Faculty of Health Sciences, University of the Witwatersrand, Johannesburg, South Africa

South Africa has the largest number of children living with HIV (CLHIV) globally, with most untreated CLHIV being 5 years and older. Despite efforts to close this gap, including prioritising paediatric case finding, many CLHIV 5–14 years are not in care.

We aimed to review programme and research data and compare these with model estimates to better understand this gap in Johannesburg, one of the districts with the highest number of CLHIV in South Africa.

We included CLHIV 5–14-year-olds in Johannesburg. Naomi model estimates were compared with case finding data from the District Health Information System and universal HIV testing data from 10 primary health care clinics. From Naomi, the number of CLHIV, HIV prevalence, and number on antiretroviral treatment (ART) were used. HIV test and test positive data were used from the two sources (September 2020 to July 2021).

According to the Naomi model, in September 2020, there were an estimated 13 350 CLHIV 5 to 14 years, of whom 46.8% were on ART (6258). Of those not on ART, 36.9% had never been tested for HIV (2620). Johannesburg had an HIV prevalence of 1.6% in children 5–14 years, with an untreated prevalence of 0.9%. Routine data over a period of 12 months indicate that 54 528 were tested finding 523 HIV-positive children, a yield of 1.0%. Data representing universal testing elicited a lower yield of 0.3% (3595 children tested with 11 testing positive in 10 weeks).

Case finding is becoming increasingly difficult with decreasing yields, especially when non-targeted or universal testing is conducted at health facilities. Expansion of case finding strategies as well as targeted approaches are needed in this age group. Alternative strategies are needed to link CLHIV with known status but not on ART into care, and retain those already on ART, if the untreated gap is going to improve.

The authors would like to thank the Johannesburg Health District management and facility teams and the Anova teams for their dedication to caring for children living with HIV.

**Ethical considerations:** Permission has been granted to conduct this analysis through the Human Sciences Research Council Ethics Committee under the ethics approval number REC 3/22/08/18.

**Funding information:** The Anova Health Institute non-profit company is supported by the United States President’s Emergency Plan (PEPFAR) programme via the United States Agency for International Development (USAID) under the Cooperative Agreement number AID-674-A-12-00015.

